# Maternal Nutrition Status Associated with Pregnancy-Related Adverse Outcomes

**DOI:** 10.3390/nu13072398

**Published:** 2021-07-13

**Authors:** Maria J Miele, Renato T Souza, Iracema M Calderon, Francisco E Feitosa, Debora F Leite, Edilberto A Rocha Filho, Janete Vettorazzi, Jussara Mayrink, Karayna G Fernandes, Matias C Vieira, Rodolfo C Pacagnella, José G. Cecatti

**Affiliations:** 1 Department of Obstetrics and Gynecology, School of Medicine, University of Campinas (UNICAMP), Campinas 13083-881, SP, Brazil; miele.mjo@gmail.com (M.J.M.); renatotsouzasp@gmail.com (R.T.S.); deborafariasleite@gmail.com (D.F.L.); jussaramayrink@gmail.com (J.M.); karayna@gmail.com (K.G.F.); matias.vieira@kcl.ac.uk (M.C.V.); rodolfopacagnella@gmail.com (R.C.P.); 2 Department of Gynecology and Obstetrics, Botucatu Medical School, Sao Paulo State University (Unesp), Botucatu 18618-970, SP, Brazil; iracema.calderon@gmail.com; 3 MEAC–Maternity School of the Federal University of Ceará, Fortaleza 60430-270, CE, Brazil; edson.lucena@hotmail.com; 4 Department of Maternal and Child Health, Federal University of Pernambuco, Recife 50670-901, PE, Brazil; edilbertorocha@globo.com; 5 Department of Obstetrics and Gynecology, Maternity Hospital, Federal University of RS, Porto Alegre 90035-903, RS, Brazil; janetegestarbem@gmail.com; 6 Department of Gynecology and Obstetrics, Jundiai School of Medicine, Jundiaí 13202-550, SP, Brazil; 7 Division of Women and Children’s Health, School of Life Course Sciences, Faculty of Life Sciences and Medicine, London SE1 7EH, UK

**Keywords:** maternal nutrition, preterm birth, preeclampsia, small-for-gestational-age, gestational diabetes mellitus

## Abstract

Although maternal nutrition has an impact on fetal development and gestational outcome, tracking maternal nutrition in outpatient practice is still complex and involves proper technical capacitation in this area. Nevertheless, the association between nutritional variables may broaden the ability to predict the occurrence of gestational disorders and prevention management. We aimed to identify factors that could indicate the probability of adverse outcomes in mid-pregnancy. From a cohort of 1165 nulliparous pregnant women without any previous disease, the nutritional status was assessed by body mass index (BMI) and mid-upper arm circumference (MUAC), associated with dietary patterns and sociodemographic characteristics. Two predictive models with nutritional status for screening the occurrence of adverse outcomes of preterm birth, gestational diabetes mellitus, small-for-gestational-age newborns and preeclampsia were developed. The odds of adverse outcomes were higher in non-white (*p* < 0.05) obese women and with high protein consumption. There was no significant difference between the models, with an overall accuracy of 63% for both models and a probability of success in predicting adverse outcomes (BMI = 61%, MUAC = 52%). This study of Brazilian pregnant nulliparous women offers two possible options for early tracking of adverse gestational outcomes that should be further externally validated.

## 1. Introduction

During pregnancy, there is a major increase in a women’s physiological demand, and to meet these requirements, healthy eating habits must be encouraged. However, the phenomenon called “nutritional transition” influenced by a major consumption of food with a high density of calories and poor in micronutrients triggers dual consequences of malnutrition and obesity [1]. Meal patterns rich in refined carbohydrates, fats and sweets increase the risk of gestational diabetes mellitus (GDM), which is associated with preterm birth (PTB) [2]. Moreover, inadequate dietary habits affect women’s health, worsening hypertensive disorders, leading to preeclampsia (PE) and the birth of newborns that are small-for-gestational-age (SGA) [3]. Although nutritional follow-up is a simple action to prevent and reduce disorders, the assessment of factors related to maternal nutrition in less-resourced settings is a complex activity since it must consider access to food, regional culinary and body composition, as well as sociodemographic characteristics and local health facilities limitations [4].

Antenatal care (ANC) is a window of opportunity to track inadequacies of nutrition and health status of women, providing useful information for monitoring and prevention purposes, depending on the level of local resources [5]. The World Health Organization (WHO) guideline recommends nutrition counseling in ANC, as nutrition education, adequacy of daily energy and protein intake, micronutrient supplements, physical activity and preventing excessive weight gain during pregnancy [6]. For this purpose, the WHO recommends nutrition training for health professionals to assess nutrition conditions among pregnant women [7].

To assess nutrition status, most frequently, the body mass index (BMI) is calculated using the pre-pregnancy weight, which has a fundamental role in determining the total amount of weight gain, monitoring gestational development and providing nutritional counseling [8]. However, when information about pre-pregnancy weight is missing, the health system in Brazil recommends the charts of Atalah according to gestational week [9,10]. Furthermore, in low or middle-income countries, it is common for women to seek antenatal care late, without information about the previous nutritional status [11,12].

Moreover, BMI, as the only factor associated with maternal nutrition, is unable to provide all the answers that trigger an adverse condition in the pregnant woman and her offspring. This information needs to be associated with anthropometric data and sociodemographic conditions, involving diverse dietary habits that bring a wealth of knowledge to the decision-making process [13,14].

In the year 2015, more than 300 thousand women died from pregnancy-related complications, and over 2 million infants were stillborn. These adverse results could be minimized during prenatal care by risk identification, health promotion and diagnosis of preventable disorders [15]. In locations with underprivileged structures, deaths and damages increase exponentially. Therefore, the use of a predictive model to identify potential chances could theoretically modify this scenario. Using information from the profile of a diverse sample, this analysis aimed to identify clinical, sociodemographic and nutritional factors by different anthropometric tools related to risks of adverse gestational outcomes and develop a model capable of predicting the chance of these outcomes, of facilitate tracking of cases and counseling of pregnant women still in mid-pregnancy, on time to possibly reduce health risks.

## 2. Materials and Methods

### 2.1. Study Design

**Figure 1:**
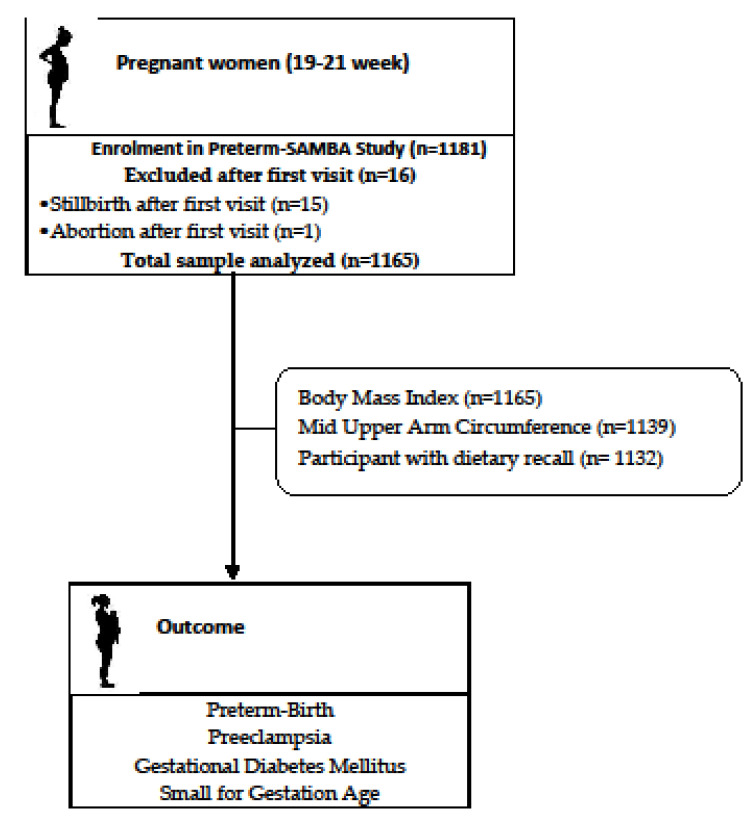
Flow chart of the study sample.

This analysis addresses the secondary objectives of the multicenter cohort study titled: “Preterm SAMBA–Preterm Screening and Metabolomics in Brazil and Auckland” [16], with an analytical approach of a nested case-control design. Pregnant women with singleton pregnancies were included from 2015 to 2018 in five public obstetric referral hospitals, located in three geographical regions and with demographic characteristics that best represented the diversity of social/ethnic aspects and dietary habits in the Northeast, South and Southeast of Brazil [17]. The flow chart of the study sample is shown in Figure 1. All women included in this study were nulliparous, without a history of previous severe disease, with gestational age confirmed by early ultrasound and gestational age at inclusion in the study between 19 and 21 weeks. Details of the study methods and procedures are available in a previous publication [16].

All women signed an individual informed consent before entering the study. The Preterm-SAMBA study followed the ethical principles of the Declaration of Helsinki (2013). It was approved by the Research Ethics Committee of all participating centers (protocol of the coordinating center 20182318.8.0000.5404), in addition to the National Research Ethics Committee (CONEP).

### 2.2. Data Collection

All nulliparous pregnant women considered to be at low risk and between the 19th and 21st weeks of gestation were invited to participate in this study. Those with a previous history of three abortions, cervical alterations, major fetal anomaly, Mullerian anomalies, history of scalpel cervical conization, chronic corticosteroid use and detected or self-reported preexisting disease including hypertensive disorder, previous diagnosis of diabetes mellitus, renal disease, systemic lupus erythematosus or antiphospholipid syndrome, sickle cell anemia and HIV positive serology, were excluded from the study. Women taking medication or supplementation that could interfere in the outcome assessment, such as aspirin, calcium, fish oil, vitamin C, vitamin E or heparin, were also excluded. Women following the Brazilian Health System guideline recommendations for supplements during pregnancy, such as folic acid, iron or a multivitamin, were not excluded, and they were identified with the questionnaire.

For nutritional status assessment, all centers used an electronic scale and duly calibrated anthropometer, and measurements were taken at the time of study entry. In all hospitals, trained staff of the healthcare team made the anthropometric measurements of the woman (weight, height and arm circumference); all these measurements were taken three times and recorded as rounded values according to standardized criteria defined by the Food and Nutritional Surveillance System of the Ministry of Health [18]. Body mass index (BMI) was automatically calculated by software from the electronic platform of the study, using weight and height measurements. Categories of anthropometric measures were used based on criteria of the Brazilian Ministry of Health, which follows the Atalah curve [10]. Measurement of mid-upper arm circumference (MUAC) was made on the left arm after marking the midpoint between the olecranon and the acromion process by using a non-elastic tape. To define the cutoff point for each MUAC category, correlation, sensitivity and specificity tests were applied, with results of the evaluation of measurements taken at three time points during pregnancy. MUAC categories as defined by cutoff point measures (cm) were: Obese >30.15, Overweight 28.11–30.15, Adequate: 25.75–28.10, and Underweight, <25.75 [19].

Sociodemographic data were self-reported. All collected data were inserted into an electronic platform (MedSciNet^®^ AB, Sweden). To compound the profile of dietary patterns, one 24 h diet recall (R24 h) was applied, which, according to Willett (2012), is adequate to define an eating habit profile [20]. The questionnaire was applied at the time the woman entered the study (19–21 weeks) by healthcare professionals trained by a dietitian, using the multi-step method, which is a standardized process oriented by steps to stimulating the respondent’s memory and increasing the accuracy of the respondent’s information [21]. Serving size was estimated in a household measure and was based on kitchen utensil photographs and food size characterized as small, medium and large according to the Brazilian Ministry of Health [22]. To standardize servings, household measures were converted into grams or milliliters of consumption using Brazilian and international reference manuals [23,24,25]. Information on industrial food labels and details of culinary recipes were also used.

Foods were grouped according to nutritional characteristics and degree of industrial processing using the NOVA classification [26]. The principal components analysis (PCA) technique was applied, with varimax orthogonal rotation [27], to identify food patterns and reduce diet variability. Five food patterns were identified by PCA: “Obesogenic” with a greater representation of ultra-processed and processed foods composed of refined carbohydrate, fats and sweets; “Traditional” mostly composed of natural or minimally processed foods in addition to beans, meats and eggs; “Intermediate” represented by a lower amount of consumption, but containing the same characteristics as the Obesogenic pattern; “Vegetarian” with a diet rich in dairy products, fruits and vegetables; and “Protein” with a predominance of protein foods with kinds of fatty meats, eggs and beans, and a very low quantity of natural foods [28]. The predominant dietary patterns of women were categorized and used in this study as a variable of quality.

The definition of cases of preeclampsia (PE) and gestational diabetes mellitus (GDM) was based on international criteria [29,30]. Preterm births (PTB) were considered for all women who gave birth before reaching 37 weeks of pregnancy [31]. The definition of small-for-gestational-age (SGA) newborns, according to the 10th percentile (<p10) adjusted for maternal characteristics (ethnicity, weight, height and parity), gestational age at birth and infant sex was performed using the GROW centile calculator: https://www.gestation.net/GROW_documentation.pdf (accessed on 19 May 2021) [32]. The variable named adverse pregnancy outcome (APO) was defined as the presence of at least one of the following conditions: PE, GDM, PTB or SGA. Initially, the sociodemographic and nutritional variables of women were described, and then two groups were created and categorized according to the occurrence of some outcome (PE, GDM, PTB, SGA).

### 2.3. Statistical Analysis

For an initial exploration, univariate analysis related to nutritional selected variables and the estimated risk of any occurrence of APO were conducted using odds ratios. The prediction model was developed in steps after different combinations of factors based on clinical criteria and analyzing each result of the adjustment model. The multiple logistic regression using the generalized linear model analyses were conducted using independent variables as a predictor for the adverse results (dependent variables). The coefficients were estimated from the data using the maximum likelihood method, maximizing the probability of the outcome occurring and a predictive accuracy test. The effect coefficients exert on the chance of the adverse event occurring was observed according to the positive (indicating greater chance) or negative (indicating protective effect) value. The estimated significance of coefficients has been tested by the Wald statistic. Analysis of residual values by the regression of minimal squares was conducted, applying Cook’s distance. To measure multicollinearity, the variance inflation factors (VIF) were examined, and values >5 were considered inadequate. For each model, the BMI and MUAC anthropometric variables were tested, aimed at confirming which assessment tool was a better model predictor for adverse gestational outcomes. To compare the quality of both models, we used the Akaike information criterion (AIC). The performance of the final multivariable model to predict the outcome was tested with the overall accuracy. The results of these analyses are presented using the odds ratio (OR) and 95% confidence interval (95% CI). For the remaining analyses, *p*-values <0.05 were considered significant. Logistic regression analyses used the library packages “Pac-Man” and “sjPlot” of the R Core Team software (2020) [33]. This article followed the guidelines of strengthening the reporting of observational studies in epidemiology (STROBE) [34].

## 3. Results

The majority of women in the sample were non-white, had a low-income and low-schooling level. Although the majority of women had an adequate BMI, calculated at the time of study entry (19–21 weeks of gestation), the MUAC measurements showed that most of these women were situated at the extremes of classifications, with excess or insufficient arm circumference measurements (Table 1).

Table 2 shows the analyses of variables associated with the diagnosis of PTB, SGA, GDM and PE. The results demonstrated an association between excess weight with unfavorable outcomes of PE and GDM. Women from the northeastern region had higher chances of developing PE. A diet rich in protein increased the probability of developing preeclampsia and PTB. Whereas women of color/ethnic non-white are the most common factors for SGA and DGM.

**Figure 2:**
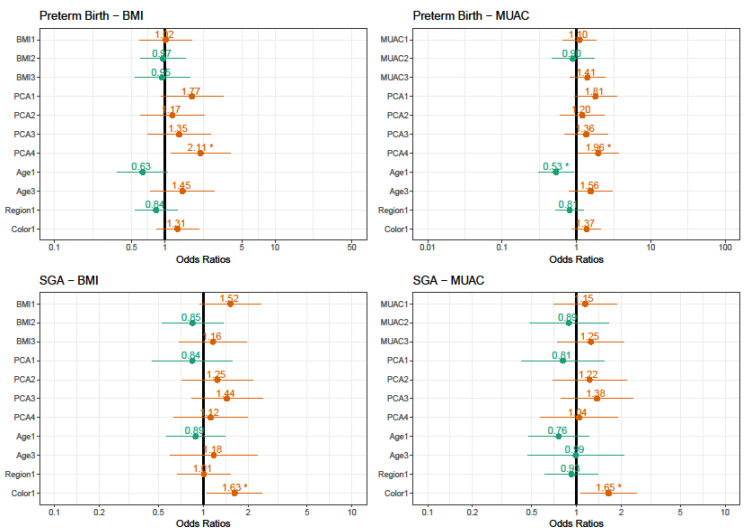
Estimated risks for Preterm-birth and small-for-gestational-age using BMI or MUAC. BMI 1: obese; BMI 2: overweight; BMI 4: underweight. MUAC 1: obese; MUAC 2: overweight; MUAC 4: underweight. PCA 1: Obesogenic; PCA 3: Intermediate; PCA 4: Vegetarian; PCA 5: Protein. Age 1: <20, Age 3: >34 years; Region 1: Northeast. Color 1: Non-white. Preterm-birth AIC = BMI model: 782.8931/MUAC model: 760.9102. Small-for-gestational-age AIC = BMI model: 860.8467/MUAC model: 829.7310. * Values of OR are significant at *p* < 0.05.

Figure 2 shows the results of models of multivariate analyses adjusted for preterm birth and small-for-gestational-age newborns. The intention was to compare the two options of anthropometric measurements and predicted chances for the occurrence of preterm birth and small-for-gestational-age newborns. For the PTB, the protein diet patterns have double the chance of this condition. When we analyzed the overall effect for the MUAC model, age was a predictor for PTB (χ^2^ = 7.8115, *p* = 0.020). While for SGA the coefficients for color/ethnicity (non-white) showed increased chances for SGA (χ^2^ = 5.0759, *p* = 0.024).

**Figure 3:**
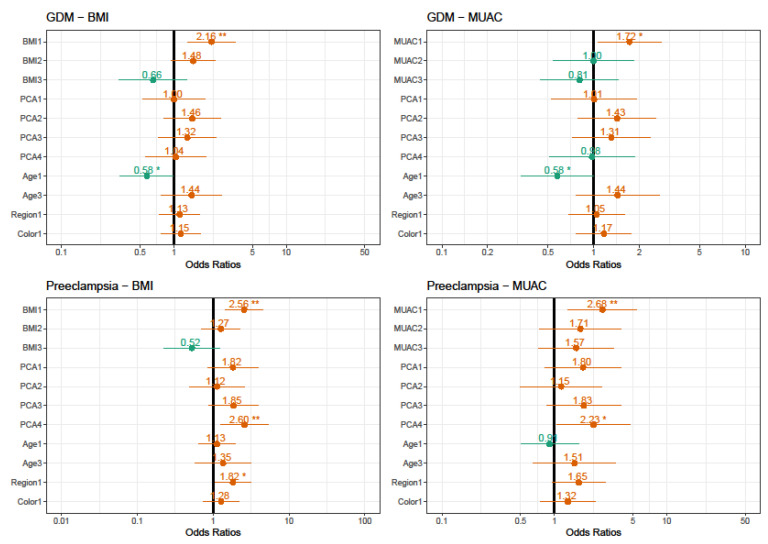
Estimated risks for Gestational diabetes mellitus and preeclampsia using BMI or MUAC. BMI 1: obese; BMI 2: overweight; BMI 4: underweight. MUAC 1: obese; MUAC 2: overweight; MUAC 4: underweight. PCA 1: Obesogenic; PCA 3: Intermediate; PCA 4: Vegetarian; PCA 5: Protein. Age 1: <20, Age 3: >34 years; Region 1: Northeast. Color 1: Non-white. Gestational diabetes mellitus AIC = BMI model: 789.3831/MUAC model: 776.5544. Preeclampsia AIC = BMI model: 589.2154/MUAC model: 574.0860. * Values of OR are significant at *p* < 0.05. ** Values of OR are significant at *p* < 0.01.

Figure 3 shows the estimated adjusted risks for GDM and PE, differentiating the anthropometric measurement used between each adjusted analysis. We can observe, in common, obesity increasing the odds and showing as a good predictor for all analyses in BMI models (χ^2^ = 15.2024, *p* < 0.001) and MUAC (χ^2^ = 10.7377, *p* < 0.013) for GDM. The same relevance for the odds and predictor was obtained for PE by the BMI model (χ^2^ = 16.3289, *p* < 0.001) or MUAC (χ^2^ = 8.7479, *p* < 0.032). However, they were different in those younger ages as showed a protective factor for the development of GDM, and protein diet patterns double the chances for PE in both models. While the model using the BMI has shown women from the Northeast region seem to have higher risks for this condition, in each of the models analyzed, the difference between the anthropometric measurements of BMI and MUAC, according to the Akaike information criterion (AIC) was very tiny, which is under the principle of parsimony.

**Figure 4:**
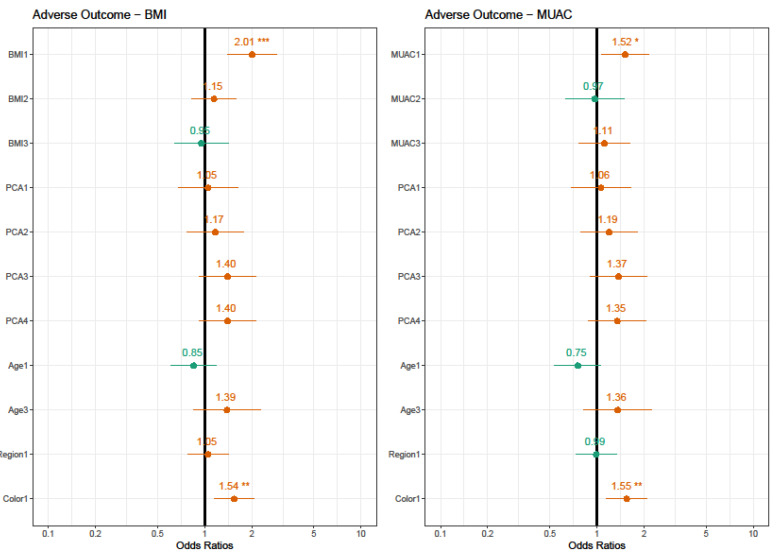
Estimated risks of adverse pregnancy outcome using BMI or MUAC. BMI 1: obese; BMI 2: overweight; BMI 4: underweight. MUAC 1: obese; MUAC 2: overweight; MUAC 4: underweight. Color 1: Non-white. AIC = BMI model: 1357.881/MUAC model: 1338.214. * Values of OR are significant at *p* < 0.05. ** Values of OR are significant at *p* < 0.01. *** Values of OR are significant at *p* < 0.001.

Figure 4 graphically shows the result of the construction of adjusted multivariate regression models that evaluated the estimated risks of all the adverse outcomes related to variables occurring. The final model had at least one significant result for any outcome explored in univariate analysis. This grouping allows us to compare the behavior of the variations, according to food pattern characteristics, region and ethnicity, in comparison to the anthropometric category measured in both forms of body composition evaluation. Thus, two models were created, one containing BMI measurement and the other containing MUAC measurement. The results showed that color/ethnicity was the factor with the largest chance to be associated with APO, evaluated by the BMI model (χ^2^ = 8.2615, *p* < 0.004) and MUAC model (χ^2^ = 8.3333, *p* < 0.003). Yet, obesity was also identified as a good predictor using both anthropometric tools, with the BMI model (χ^2^ = 15.8267, *p* < 0.001) or MUAC (χ^2^ = 7.9062, *p* < 0.047). Then we made the same evaluation for each separate outcome concerning the variables selected from this first general model.

The focus of the current study was the distinction between attributions of gestation outcomes. Considering the assumption of the overlap in underlying causes for adversities during gestation and the outcomes related among them. The probabilistic prediction model tested different attribution factors relevant to each outcome. The final assumption model is shown in Table 3, where the factors that resulted in higher risks for any adverse outcomes, by BMI or MUAC, were selected. The model predicts the probability for all adverse outcomes. The values beginning in the intercept and the independent variables with any significance for chances of occurring at least one adverse event were evaluated.

## 4. Discussion

This study combined dietary patterns, body composition and sociodemographic characteristics, showing that the combination of these factors may alert to the need for health promotion and the prevention of adverse conditions predicted by these grouped factors. The combined factors offer a final model using different anthropometric tools, showing a 61% and 52% chance of predicting the occurrence of adverse outcomes, and may help in making clinical decisions in the prenatal period. For this analysis, women were rigorously selected from a group of low-risk, nulliparous women without any severe disease to reduce the chance of biases in the identification of adverse outcomes in pregnancy. We created a predictive model that was capable of tracking the odds of adverse outcomes using a simple tool, without the need for previous training and in settings with limited resources.

A study of intervention strategies for pregnant women using two different theories resulted in 66% of the global variance in healthy eating intention and 3.4% in adherence to food group recommendations [35]. For nutrition, a mix of factors can be related to an eating habit. This manuscript showed that younger maternal age was identified as a protective factor, whereas the older were more likely to develop unfavorable health conditions. One study with low-income pregnant women tested the relationships involving distress, eating habits and maternal age, resulting in 19% of the factors explained for the dietary choices [36].

The development of a pathological condition is multifactorial, and variables that trigger the disorder are not always the same, requiring individualized evaluation. However, separate evaluation of each outcome may conceal the associations between these outcomes, as occurs in pregnant women who develop preeclampsia and give birth to small-for-gestational-age babies or women with worsening of gestational diabetes mellitus who require a therapeutic preterm birth [37,38].

To date, the assessment of nutritional status using the BMI is widely applied to investigate the odds of a negative outcome. Nevertheless, a review of 5874 studies compared the World Health Organization recommendations and showed that BMI alone was not associated with a higher probability of adverse results [39]. In contrast, one study of low-income pregnant women associated pre-pregnancy BMI with maternal diets and obtained a 19% variance for maternal nutritional adequacy. However, when BMI was associated with maternal age and nutrition information, the model has predicted the weight at birth at a rate of 52% [40]. Another study compared ethnic factors associated with the incidence of GDM among western and eastern pregnant women. The authors associated obesity, excess weight gain, diets and lifestyles as the main causes of glucose intolerance [41].

In our results, there were two-fold odds of developing GDM in obesity associated with both tools that measure body composition. For the same outcome, when each variable was analyzed individually, only non-white ethnicity showed a significant result. The data on age is in agreement with a recent review and metanalysis with over 120 million women, reinforcing the information that the incidence of GDM increases linearly with increasing age [42].

The anthropometric classification of obesity had a greater impact on PE and GDM, in contrast to PTB and SGA. For PE, the results remained similar to obesity; women that consumed a diet richer in protein showed higher odds of having arterial blood pressure disorder. Associated with these factors, the model using the BMI reveals the Northeast had double chances of PE development. In addition to other factors, Northeastern cuisine may have led to these results, potentiating this outcome. A diet consisting of red meat and cheese may contain a large amount of sodium and saturated fat, including some typical regional foods such as “carne de sol” or “sun-dried meat” or “baião de dois” and “feijoada” (the former is a mixture of beans, rice and sausage and the latter is a bean and pork stew) are widely consumed and are part of the regional cuisine [43]. The same results were obtained by a study that analyzed household food insecurity in Brazilian through a statistical prediction model. According to the authors, the model showed a strong predictive capacity power to estimate the Northeast with 65% higher chances of food insecurity [44].

A similar result was obtained with data of 66,651 pregnant women from the Danish national birth cohort, with no significance between food patterns and increased arterial blood pressure. Nevertheless, sodium consumption in milligrams showed a risk of 54% for increased arterial blood pressure and 20% for the development of preeclampsia [45]. In addition to PE outcome, our study showed that women with a higher protein-rich diet have a twofold chance of having PTB.

Another advantage of this study is to offer two options of using different anthropometric tools to rapidly track body composition categories and the risk of undesirable outcomes. Often the first prenatal consultations are scheduled late, resulting in a lack of information about baseline or pre-gestational weight. In other cases, there is a loss in follow-up consultations, and weight data are lost. These barriers may hinder weight assessment and patient follow-up for BMI calculation [46]. Historically, in many low- and middle-income countries or in those under emergencies, the MUAC has been adopted as an alternative to weight measurement for initial screening of an undesirable health event [47]. In locations with limited resources, MUAC is a substitute for BMI, where values lower than 23 cm point to the risk of SGA, and higher values (>33 cm) may be used to track PE and GDM [48].

This study has some limitations. This is an ancillary analysis from data derived from the main study focusing on measured physical activity and sleep patterns as already informed. Therefore, some important nutritional information was not available as ideally recommended. In addition, taking into account the late initiation of prenatal care, some important information on pre-pregnancy weight and weight gain could not be tracked. We relied on information from an anthropometric assessment performed at mid-pregnancy and a single 24 h diet record from the same period, without considering possible changes in eating habits alongside pregnancy.

There is still no rule that defines the perfect percentage in a predictive statistical model. This is an exploratory study, and based on this model, more probabilistic tests must be done to confirm the usefulness of this predictive model. The strength of this study is the percentage of probability of success in predicting outcomes in using models for early screening of women with a tendency to develop complications during pregnancy. These models can support clinical decision-making. A limitation of the study is that the main investigation was not initially designed for this analysis, suggesting that further tests are needed in future studies to externally validate and confirm the utility of the models proposed here.

## 5. Conclusions

The combination of factors related to food patterns and one anthropometric tool such as the MUAC or BMI is useful in early clinical evaluation and may be applied to support clinical decision-making in tracking women most likely to develop an adverse obstetric or neonatal condition.

## Figures and Tables

**Table 1:** Sample characterization according to sociodemographic and nutritional characteristics of pregnant women.

Maternal Features #	*n* = 1165	%
**^a^ BMI (kg/m^2^)**		
Obese	199	17.1
Overweight	299	25.7
Adequate	461	39.6
Underweight	205	17.6
**^b^ MUAC (cm)**		
Obese	366	32.2
Overweight	180	15.8
Adequate	281	24.7
Underweight	310	27.3
**^c^ Dietary Patterns**		
Obesogenic	197	17.4
Traditional	241	21.3
Intermediate	242	21.4
Vegetarian	233	20.6
Protein	219	19.3
**Income (per year)**		
≤12,000(USD)	861	73.9
>12,000(USD)	304	26.1
**Occupation**		
Working	585	50.2
Not working	580	49.8
**Age (years)**		
≤19	291	25.0
20–35	796	68.3
>35	78	6.7
**Education (years)**		
<12	791	67.9
≥12	374	32.1
**Region**		
Northwest	565	48.5
South/Southwest	600	51.5
**Color/ethnicity**		
White	462	39.7
Non-white	703	60.3
**Gestational Outcome**		
Preterm birth	125	10.7
^d^ Small for Gestation Age	146	12.7
^e^ Gestational Diabetes Mellitus	142	14.1
Preeclampsia	87	7.5
^f^ Global adverse outcome	401	39.0

Missing information for: ^a^: 1, ^b^: 28, ^c^: 33, ^d^: 13, ^e^: 157, ^f^: 137. # Measured during the first study visit of antenatal care (19–21 weeks of gestation). MUAC: mid-upper arm circumference.

**Table 2:** Estimated risk of adverse gestational outcomes according to maternal sociodemographic and nutritional variables.

Factors	PTB	SGA	GDM	PE
**^a^ BMI (kg/m^2^)**				
Obese	1.07(0.63–1.80)	1.53(0.95–2.43)	**2.40**(**1.50–3.83**)	**2.43**(**1.39–4.25**)
Overweight	0.99(0.61–1.57)	0.88(0.55–1.41)	**1.65**(**1.05–2.58**)	**1.29**(**1.39–4.25**)
Adequate	1.00	1.00	1.00	1.00
Underweight	0.89(0.50–1.51)	1.22(0.74–1.98)	0.56(0.27–1.08)	0.71(0.31–1.48)
**^b^ MUAC** (**cm**)				
Obese	1.12(0.67–1.90)	1.19(0.73–1.94)	**1.85(1.17–2.99)**	**2.84(1.47–5.94)**
Overweight	0.98(0.51–1.84)	0.95(0.51–1.72)	1.05(0.57–1.91)	1.91(0.84–4.45)
Adequate	1.00	1.00	1.00	1.00
Underweight	1.35(0.81–2.30)	1.24(0.76–2.06)	0.68(0.38–1.22)	1.78(0.86–3.90)
**^c^ Dietary Patterns**				
Obesogenic	1.72(0.91–3.31)	0.86(0.46–1.58)	0.96(0.51–1.81)	1.66(0.79–3.56)
Traditional	1.00	1.00	1.00	1.00
Intermediate	1.24(0.65–2.40)	1.19(0.69–2.05)	1.39(0.80–2.48)	0.92(0.40–2.06)
Vegetarian	1.49(0.79–2.85)	1.35(0.79–2.31)	1.36(0.77–2.43)	1.56(0.76–3.30)
Protein	**2.20(1.21–4.11)**	1.07(0.60–1.88)	0.99(0.54–1.83)	**2.06**(**1.03–4.28**)
**Income** (**per year**)				
≤12,000(USD)	1.25(0.81–1.98)	1.11(0.75–1.69)	1.50(0.96–2.42)	0.92(0.57–1.53)
>12,000(USD)	1.00	1.00	1.00	1.00
**Occupation**				
Working	1.00	1.00	1.00	1.00
Not working	0.69(0.47–1.00)	0.90(0.63–1.27)	1.08(0.76–1.54)	0.81(0.52–1.25)
**Age** (**year**)				
≤19	0.63(0.38–1.01)	0.95(0.63–1.43)	**0.45**(**0.26–0.74**)	0.91(0.52–1.50)
20–35	1.00	1.00	1.00	1.00
>34	1.41(0.70–2.62)	1.13(0.55–2.13)	1.40(0.74–2.51)	1.21(0.49–2.58)
**Education** (**year**)				
<12	1.01(0.68–1.51)	1.00(0.69–1.46)	0.98(0.67–1.43)	0.94(0.60–1.51)
≥12	1.00	1.00	1.00	1.00
**Region**				
Northwest	0.94(0.65–1.37)	1.10(0.77–1.55)	1.03(0.72–1.46)	**1.82**(**1.16–2.87**)
South/Southwest	1.00	1.00	1.00	1.00
**Color/ethnic**				
White	1.00	1.00	1.00	1.00
Non-white	1.14(0.78–1.69)	**1.47**(**1.02–2.14**)	**1.02**(**0.71–1.46**)	1.50(0.95–2.44)

The results by odds ratio and confidence interval 95%. MUAC: mid-upper arm circumference; PTB: preterm birth; GDM: gestational diabetes mellitus; SGA: small-for-gestational-age; PE: pre-eclampsia. Missing information for: a:1(PTB), b:28(PTB); c:33(PTB) and 32(SGA); a:1 (PE), b:17 (GDM), b:28(PE), c:30(GDM) and 33(PE), a:1, b:20, c:30. Values in bold mean they are significant at *p* < 0.05.

**Table 3:** Models for the probability of success in predicting the occurrence of any adverse outcome according to maternal nutritional characteristics in mid-pregnancy.

	Equation Models	Probability	Accuracy
(1)	$\hat{y}$ = −1.05121 _β0_ + 0.69694 _β1 (BMI1)_ + 0.32953 _β3 (PCA5)_ + 0.45503 _β4 (Color1)_	61%	63%
(2)	$\hat{y}$ = −1.03799 _β0_ + 0.41557 _β1 (MUAC1)_ + 0.29974 _β2 (PCA5)_ + 0.43346 _β4 (Color1)_	52%	63%

The predicted response was calculated based on the intercept of each model and variables that respond significantly to the odds of occurring any adverse outcome. Accuracy of the overall effect to predict the outcome.

## Data Availability

The data presented in this study are available on reasonable request from the corresponding author. The data are not publicly available due to privacy and ethical restrictions.
